# Differences in incidence, nature of symptoms, and duration of long COVID among hospitalised migrant and non-migrant patients in the Netherlands: a retrospective cohort study

**DOI:** 10.1016/j.lanepe.2023.100630

**Published:** 2023-04-07

**Authors:** Felix Patience Chilunga, Brent Appelman, Michele van Vugt, Kirsten Kalverda, Patrick Smeele, Josien van Es, Willem Joost Wiersinga, Mikael Rostila, Maria Prins, Karien Stronks, Marie Norredam, Charles Agyemang

**Affiliations:** a Department of Public and Occupational Health, Amsterdam Public Health, University of Amsterdam Medical Centers, University of Amsterdam, Amsterdam, The Netherlands; b Center for Experimental and Molecular Medicine, Amsterdam University Medical Centers, Amsterdam Institute for Infection and Immunity (AII), University of Amsterdam, Amsterdam, The Netherlands; c Division of Infectious Diseases, Amsterdam University Medical Centers, Location AMC, Amsterdam Institute for Infection and Immunity (AII), University of Amsterdam, Amsterdam Public Health, Amsterdam, The Netherlands; d Department Pulmonary Medicine, Amsterdam UMC, Location AMC, University of Amsterdam, Amsterdam, The Netherlands; e Department of Public Health Sciences, Stockholm University, Stockholm, Sweden; f Centre for Health Equity Studies, Stockholm University/Karolinska Institutet, Stockholm, Sweden; g Department of Infectious Diseases, Public Health Service of Amsterdam (GGD), Amsterdam, The Netherlands; h Centre for Urban Mental Health, University of Amsterdam, Amsterdam, The Netherlands; i Section for Health Services Research, Department of Public Health, Research Centre for Migration, Ethnicity and Health (MESU), University of Copenhagen, Copenhagen, Denmark; j Department of Infectious Diseases, Copenhagen University Hospital, Amager and Hvidovre, University of Copenhagen, Copenhagen, Denmark; k Division of Endocrinology, Diabetes, and Metabolism, Department of Medicine, Johns Hopkins University School of Medicine, Baltimore, MD, USA

**Keywords:** Post-acute COVID-19 syndrome, Ethnicity, Transients and migrants

## Abstract

**Background:**

Comprehensive data on long COVID across ethnic and migrant groups are lacking. We investigated incidence, nature of symptoms, clinical predictors, and duration of long COVID among COVID-19 hospitalised patients in the Netherlands by migration background (Dutch, Turkish, Moroccan, and Surinamese origin, Others).

**Methods:**

We used COVID-19 admissions and follow up data (January 2021–July 2022) from Amsterdam University Medical Centers. We calculated long COVID incidence proportions per NICE guidelines by migration background and assessed for clinical predictors via robust Poisson regressions. We then examined associations between migration background and long COVID using robust Poisson regressions and adjusted for derived clinical predictors, and other biologically relevant factors. We also assessed long COVID symptom persistence at one-year post-discharge.

**Findings:**

1886 patients were included. 483 patients had long COVID (26%, 95% CI 24–28%) at 12 weeks post-discharge. Symptoms like dizziness, joint pain, insomnia, and headache varied by migration background. Clinical predictors of long COVID were female sex, hospital admission duration, intensive care unit admission, and receiving oxygen, or corticosteroid therapy. Long COVID risk was higher among patients with migration background than Dutch origin patients after adjustments for derived clinical predictors, age, smoking, vaccination status, comorbidities and remdesivir treatment. Only 14% of long COVID symptoms persisted at one-year post-discharge.

**Interpretation:**

There are significant differences in occurrence, nature of symptoms, and duration of long COVID by migration background. Studies assessing the spectrum of functional limitation and access to post-COVID healthcare are needed to help plan for appropriate and accessible healthcare interventions.

**Funding:**

The Amsterdam UMC COVID-19 biobank is supported by the 10.13039/100019573Amsterdam UMC Corona Research Fund and the Talud Foundation (Stichting Talud). The current analyses were supported by the 10.13039/501100009708Novo Nordisk Foundation [NNF21OC0067528].

**Research in context**

**Evidence before this study**

We used the MESH terms “Post-Acute COVID-19 Syndrome”, “Ethnicity”, and “Transients and Migrants” to search PubMed for articles published between March 1, 2020, and December 1, 2022. Two studies in the UK (one in a sample of non-hospitalized adults and the other in a combined sample of hospitalized and non-hospitalized adults) have assessed the risk of long COVID across ethnic groups as part of a broad range of socio-demographic factors. The studies, however, did not investigate the nature of symptoms, predictors, and duration of long COVID symptoms across ethnic groups. Furthermore, data on ethnic differences in long COVID among individuals hospitalized with COVID-19 (who are more likely to have COVID-19 morbidity than non-hospitalized individuals) are still lacking.

**Added value of this study**

Our findings show that hospitalized patients with a migration background are more likely than Dutch origin patients to have long COVID. Long COVID symptoms such as dizziness, joint and muscle pain, palpitations, insomnia, and headache vary by migration background. Clinical predictors of long COVID were female sex, duration of hospital admission, intensive care unit admission, receiving oxygen, or corticosteroid therapy during admission. Despite being important, vaccination status against COVID-19 was not a predictor of long COVID in our models. Except for Surinamese patients, the majority of long COVID symptoms disappear within a year of hospital discharge.

**Implications of all the available evidence**

There are significant differences in the occurrence, nature of symptoms and duration of long COVID between patients with Dutch and migration background. Our findings call for further studies that will assess the spectrum of functional limitation from long COVID, as well as the differences in access to post-COVID healthcare between migrant and non-migrant groups to help plan for appropriate and accessible healthcare interventions.

## Introduction

It is widely acknowledged that COVID-19 has long-term negative effects on health. For instance, a global meta-analysis of 33 studies found that nearly half of those who contracted COVID-19 continued to experience symptoms 3 months after onset of the disease, a condition known as “post-COVID-19 syndrome”, “post COVID-19 condition”, “post-acute sequelae of SARS-CoV-2” or “long COVID”.^1^^,^^2^ Possible mechanisms suggest both organ damage and multi-system inflammation may lead to health changes that linger after the COVID-19 illness, including respiratory, cardiac, neurological, and musculoskeletal complications.^2^, ^3^, ^4^ In this paper, we use the term “long COVID” which we define as symptoms persisting at least 12 weeks after COVID-19 illness as per National Institute for Health and Care Excellence (NICE) guideline.^5^

The burden of long COVID might differ between ethnic groups. This stems from the fact that there were significant ethnic differences in SARS-CoV-2 infection rates, as well as in COVID-19 morbidity and mortality.^6^, ^7^, ^8^, ^9^ Specifically, ethnic minority populations in high-income countries had higher COVID-19 morbidity and mortality than the majority populations which was attributed to differential exposure (e.g., working in the front line), differential vulnerability to infection (e.g., having a higher burden of underlying medical conditions), differential disease consequences (e.g., having lower access to health care) and differential effectiveness of measures (e.g., vaccine hesitancy).^10^ Despite a theoretically plausible higher risk of long COVID among ethnic minority populations than majority populations, studies examining the risk of long COVID across ethnic groups have been contradictory. For instance, a study in non-hospitalized adults in the UK showed that black, and mixed population groups had higher long COVID risk than the white population.^11^ On the other hand, another study utilizing data from ten UK longitudinal study studies in a combined sample of hospitalised and non-hospitalised adults showed that South Asian and black population groups had lower odds of long COVID relative to the white population.^12^

While the two studies assessed the risk of long COVID across ethnic groups as part of a broad range of socio-demographic factors,^11^^,^^12^ the studies did not go in-depth to dissect the nature of symptoms, duration of symptoms (e.g., persistence of symptoms at one year) and predictors of long COVID symptoms across these ethnic groups. We hypothesize that on top of ethnic differences in long COVID risk, the nature, severity, predictors, and duration of long COVID symptoms will also differ across ethnic groups due to variation in factors like underlying medical conditions, and access to health care. Moreover, ethnic differences in long COVID among hospitalized individuals (who are likely to have greater COVID-19 morbidity than non-hospitalized individuals) are also still unknown.

In the Netherlands, the standard classification of ethnic groups is based on country of birth and is further classified into Dutch origin vs population with migration background.^13^ We therefore used this standard classification and investigated incidence, nature of symptoms, clinical predictors, and duration of long COVID among patients hospitalised for COVID-19 in Amsterdam (the Netherlands) by migration background.

## Methods

### Study population and design

By means of electronic patient records, we conducted a retrospective cohort study among individuals admitted to Amsterdam University Medical Centers (Amsterdam UMC) for COVID-19 and then followed up in the post-COVID clinic after discharge. The Amsterdam UMC is one of the largest hospitals in the Netherlands and a university teaching hospital (https://www.amc.nl/web/home.htm). The hospital provides care for the diverse (multi-ethnic) population of Amsterdam, making it possible to study the long-term health effects of COVID-19 across ethnic groups. Data on COVID-19 admissions were accessed from January 1 through December 31, 2021, whereas information on post-COVID clinic visits was accessed from January 1, 2021, through June 30, 2022.^14^ All admitted individuals (age ≥ 18 years old) with either a confirmed positive SARS-CoV-2 polymerase chain reaction or high clinical suspicion for COVID-19, based on clinical presentation and computed tomography imaging of the chest (COVID-19 Reporting and Data System [CO-RADS] score 4 or 5), and discharged alive were eligible for inclusion in the study. From this group, only those who attended the post-COVID clinic for follow-up care at 12 weeks post-discharge, as well as those who skipped the appointment because they felt recovered at 12 weeks post-discharge (i.e., reported to have no lingering COVID-19 symptoms) were included. Individuals who died before the post-COVID clinic appointment at 12 weeks post-discharge or those that did not show up at their post-COVID clinic appointment at 12 weeks post-discharge and gave no reason were excluded. Individuals who moved to another hospital/clinic for post-COVID follow up at 12 weeks post-discharge were also excluded.

### Ethical approval

The medical ethics committees of the Amsterdam UMC approved the study protocol, as well as the access to electronic medical records (Amsterdam UMC; 20.131). The opt-out procedure for informed consent was communicated by press release in accordance with national guidelines and the European privacy law.^15^

### Measurements

All measurements were obtained from electronic patient records. Migration background was defined according to the standard classification of Statistics Netherlands.^13^ This classification considers the country of birth of residents and their parents, thus includes immigrants and their descendants.^13^ Patients were considered of Dutch origin if: (1) they were born in the Netherlands, and at least one parent born was also born in the Netherlands or (2) they were born abroad but both their parents were born in the Netherlands. On the other hand, patients were considered to have a migration background if: (1) they were born abroad and had at least one parent born abroad (immigrants) or (2) they were born in the Netherlands, but both their parents were born abroad (immigrants’ descendants). Patients were divided into the following groups: Dutch origin, Surinamese origin, Turkish origin, Moroccan origin, other non-European origin, and unknown origin. These groups were chosen because they represent some of the main ethnic groups in Amsterdam. Patients with a Surinamese origin were further classified into South-Asian Surinamese and African Surinamese using a previously validated list based on surname.^16^

Patients were examined at the post-COVID clinic at six weeks and at twelve weeks after hospital discharge, according to standard medical practice. For periods exceeding twelve weeks, patients who still had symptoms were asked to revisit the post-COVID clinic. Long COVID was defined as symptoms persisting at least 12 weeks after discharge from hospital from COVID-19 disease as per NICE guidelines.^5^ Fatigue, dyspnoea, cough, chest pain, heart palpitations, dizziness, joint and muscle pain, loss of taste or smell, and headache were among the most frequently reported long COVID symptoms in literature and were actively evaluated at the post-COVID clinic, making them available for our research.

Demographic characteristics, comorbidities and hospital admission data including provided treatment were also obtained from the electronic hospital records. Categories of medical conditions (e.g., chronic cardiovascular diseases) and their treatments were pre-recorded. A full list of medical conditions and medications is in Appendix 1. Smoking was categorised into current smoker, former smoker, and never smoked. Underlying medical conditions were categorised into; none, one, two, three or four plus conditions.

### Statistics analyses

All data were analysed in RStudio *version 4.0.3* (R Core Team 2013, Vienna, Austria). Baseline characteristics were presented as proportions for categorical variables, as mean (SD) for normally distributed continuous variables or as median (IQR) for skewed continuous variables. Differences in baseline characteristics by migration background were tested by Kruskal Wallis tests (skewed continuous variables) and chi-square tests (for categorical variables). Nature of long COVID symptoms were assessed by migration background as proportions and visualised via Radar plots. Age and sex adjusted incidence proportions of long COVID were calculated via *DirectStandardisation package* (standardised to the structure of total sample) by migration background. One-step robust Poisson regression was used to assess clinical predictors of long COVID in the total population. Variance inflation factor (VIF) was used to assess multi-collinearity between predictor variables. Predictor variables with VIF score greater than five (highly correlated) were excluded. Robust Poisson regression models were also used to assess associations between migration background (predictor) and long COVID (outcome). Adjustments were made for clinical predictors of long COVID derived from the one-step regression, as well as for biologically relevant factors that were not significant in the one step regression (e.g., age, smoking, vaccination status against COVID-19, number of co-morbidities and receiving remdesivir treatment). Prevalence ratios (PRs) and together with 95% confidence intervals (CI) were reported. Finally, age- and sex-adjusted incidence proportions of long COVID symptom persistence at one year were calculated via *DirectStandardisation package* (standardised to the structure of total sample) by migration background. Missing values were less than 17% for all variables (Appendix 2). All missing values were imputed via Amelia II (except ethnicity whereby patients with missing ethnicity were categorised as unknown).^17^ Amelia II employs a bootstrapping-based imputation algorithm that produces essentially the same results as standard IP (imputation posterior) or EM (expectation maximization) approaches but can handle a much larger number of variables. All analyses were two-tailed at an alpha of 0.05.

### Sensitivity analyses

First, robust Poisson regression models in the main analyses were repeated with unimputed data to assess robustness of the findings. Second, robust Poisson regression models in the main analyses were performed on four separate multiple imputation datasets to verify consistency of results across multiple imputation datasets.

### Role of the funding source

The study funder had no role in the study design, data collection, data analysis, data interpretation or writing of the report. The corresponding author had full access to all the data and the final responsibility to submit for publication.

## Results

### Baseline characteristics

**Fig. 1:**
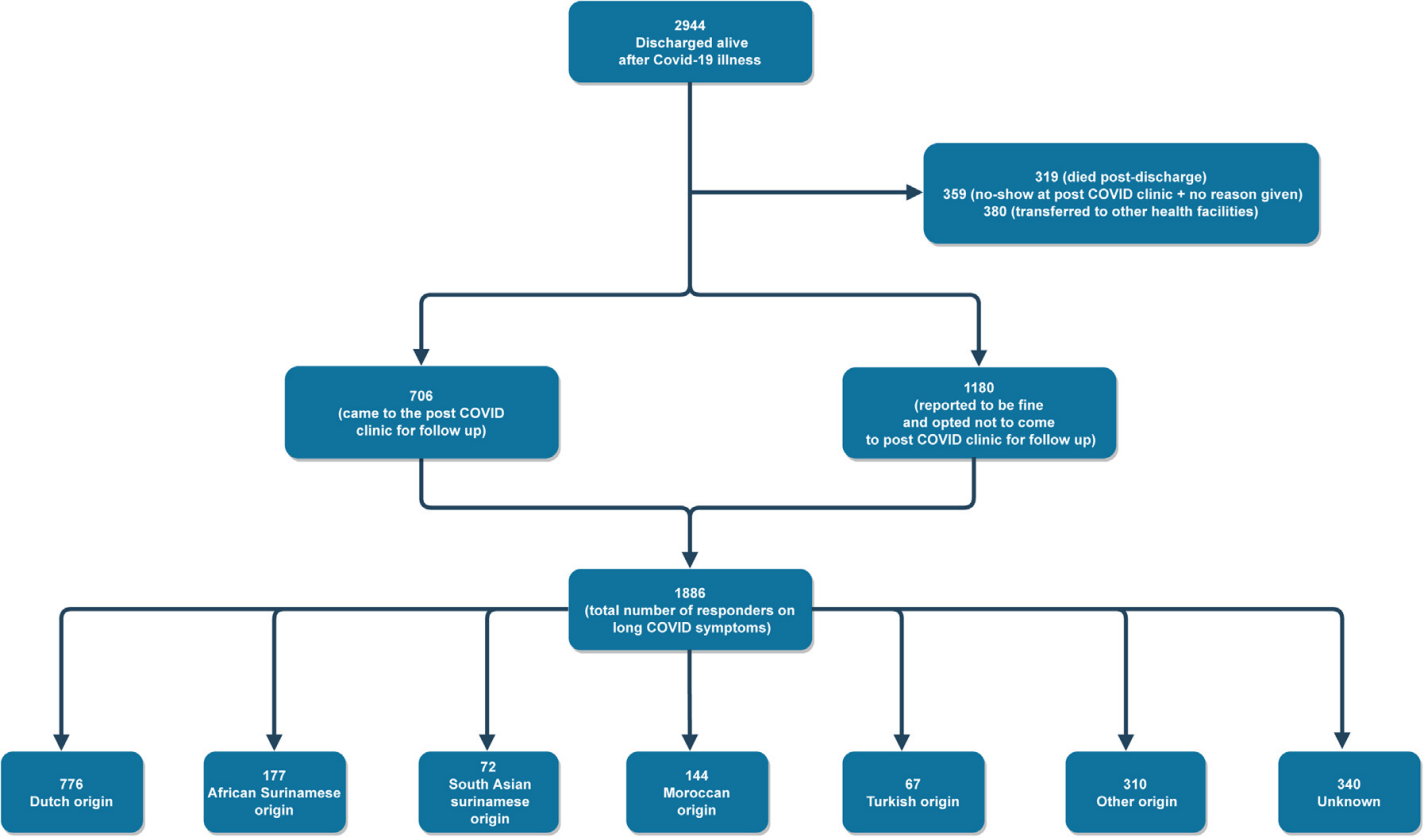
**Flow chart of participation.** Chart depicts how the final study sample was arrived at.

Between 01 January and 31 December 2021, a total of 2944 patients were discharged alive after COVID-19 hospitalisation (Fig. 1). Out of these, 319 died post-hospital discharge, 359 did not show up at the clinic and did not provide a reason, 380 were transferred to other health facilities (including to the general practitioners) hence were excluded (Fig. 1). Eventually, 1886 Patients (63%) were included in our study (Fig. 1). The included and un-included patients differed in many aspects including median age (62 vs 66 years, p < 0.001), proportion of male sex (57 vs 59%, p = 0.034), proportion of current smokers (10 vs 15%, p = 0.008), proportion of alcohol use disorder (4.6 vs 5.2%, p < 0.001), proportion of vaccination against COVID-19 (9 vs 16%, p < 0.001), proportion of obesity (28 vs 24%, p = 0.022), proportion of hypertension (35 vs 30%, p = 0.010), proportion of chronic kidney disease (10 vs 14%, p < 0.001), proportion of patients on immunesupprevise medications (8 vs 12%, p < 0.001), proportion of malignancy (5 vs 7%, p = 0.039), proportion of all chronic conditions (70 vs 65%, p < 0.001), median days of hospital admission (6 vs 8 days, p < 0.001), proportion of admission to ICU (20 vs 25%), proportion of receiving oxygen (85 vs 83%, p < 0.001), proportion of receiving corticosteroids (58 vs 48%, p < 0.001) and proportion of receiving remdesivir (10 vs 4%, p = 0.001; Table 1).

**Table 1:** Comparison of characteristics of patients included and not included in the study (imputed data).

Categories	Patients included in study N = 1886	Patients not included in study N = 1058	p-value for differences between groups
**Demographics measures**			
Migration background, n (%)			
Dutch origin	776 (41.15)	454 (42.91)	0.003
African Surinamese origin	177 (9.3)	69 (6.52)	
South Asian Surinamese origin	72 (3.82)	37 (3.49)	
Moroccan origin	144 (7.63)	70 (6.62)	
Turkish origin	67 (3.56)	34 (3.21)	
Other origins	310 (16.43)	149 (14.08)	
Unknown origin	340 (18.03)	245 (23.15)	
Age (years), median (IQR)	62 (59–71)	66 (58–66)	<0.001
Sex, n (%)			
Male	1067 (56.57)	620 (58.60)	0.034
Female	819 (43.23)	438 (41.40)	
**Behavioural measures**			
Smoking, n (%)			
Current smokers	181 (9.59)	162 (15.31)	0.008
Former smokers	621 (23.93)	536 (50.66)	
Never smoked	1084 (57.47)	360 (34.03)	
Alcohol use disorder, n (%)			
Yes	87 (4.61)	55 (5.20)	<0.001
No	1799 (95.39)	1003 (94.80)	
Vaccinated against COVID-19			
Yes	169 (8.96)	173 (16.35)	<0.001
No	1717 (91.04)	885 (83.65)	
**Underlying medical conditions**			
Obesity			
Yes	530 (28.11)	256 (24.19)	0.022
No	1356 (71.89)	802 (75.80)	
Hypertension, n (%)			
Yes	656 (34.78)	319 (30.15)	0.010
No	1230 (65.22)	739 (69.85)	
Chronic respiratory condition, n (%)			
Yes	312 (16.54)	166 (15.69)	0.547
No	1574 (83.46)	892 (84.31)	
Chronic cardiovascular condition, n (%)			
Yes	373 (19.78)	226 (21.36)	0.305
No	1513 (80.22)	832 (78.64)	
Diabetes mellitus, n (%)			
Yes	442 (23.44)	226 (21.36)	0.197
No	1444 (76.56)	832 (78.64)	
Chronic kidney disease, n (%)			
Yes	186 (9.86)	149 (14.08)	<0.001
No	1700 (90.14)	909 (85.92)	
Receiving immunosuppressive medication, n (%)			
Yes	159 (8.43)	132 (12.48)	<0.001
No	1727 (91.57)	926 (87.52)	
Chronic liver disease, n (%)			
Yes	66 (3.50)	42 (3.97)	0.514
No	1820 (96.50)	1016 (96.03)	
Malignancy, n (%)			
Yes	100 (5.30)	76 (7.18)	0.039
No	1786 (94.70)	982 (92.82)	
Chronic haematological condition, n (%)			
Yes	82 (4.35)	36 (3.40)	0.209
No	1804 (95.65)	1022 (96.60)	
Chronic neurological condition, n (%)			
Yes	190 (10.07)	108 (10.21)	0.908
No	1696 (89.93)	950 (89.79)	
Number of chronic conditions, n (%)			
None	560 (29.69)	372 (35.16)	<0.001
1 chronic condition	467 (24.76)	228 (21.55)	
2 chronic conditions	367 (19.45)	139 (13.14)	
3 chronic conditions	246 (13.04)	147 (13.89)	
4+ chronic conditions	246 (13.04)	172 (16.25)	
**Hospital admission parameters**			
Number of days admitted in hospital, median (IQR)	6.00 (3.00–11.00)	8.00 (4.00–15.00)	0.001
Rehospitalisation, n (%)			
Yes	190 (10.07)	108 (10.21)	0.001
No	1696 (89.93)	950 (89.79)	
Admitted to the ICU, n (%)			
Yes	385 (20.41)	263 (24.86)	0.005
No	1501 (79.59)	795 (75.14)	
Received oxygen, n (%)			
Yes	1608 (85.26)	880 (83.17)	0.001
No	278 (22.32)	178 (16.82)	
Received antibiotics, n (%)			
Yes	861 (45.65)	474 (44.80)	0.656
No	1025 (54.75)	584 (55.20)	
Received corticosteroids, n (%)			
Yes	1091 (57.85)	509 (48.11)	<0.001
No	795 (42.15)	549 (51.89)	
Received remdesivir, n (%)			
Yes	181 (9.60)	43 (4.06)	0.001
No	1705 (90.40)	1015 (95.94)	

**p-value for differences between groups** considered statistically significant at values less than 0.05.

**Alcohol use disorder** based on Diagnostic and Statistical Manual of Mental Disorders (DSM–5) criteria.

**Vaccinated against COVID-19** refers to receipt of any time of COVID-19 before admission irrespective of number of doses at admission.

**Obesity:** body mass index ≥30 kg/m^2^, data collected as a yes or no in the electronic medical records.

**Chronic respiratory condition:** Asthma, Alpha 1 trypsin deficiency, Asbestosis, Cryptogenic organizing pneumonia (COP), LAM, LICS, Broncho-pulmonary dysplasia or Primary ciliary dyskinesia, Bronchiectasis, Cystic fibrosis, COPD (chronic bronchitis, emphysema), Lung fibrosis, Sarcoidosis, Obstructive sleep apnoea, Pulmonary hypertension.

**Chronic cardiovascular condition:** Myocardial infarction, Cardiac arrhythmias: AVNRT, Atrial fibrillation, (supra)ventricular tachycardia, Ventricular tachycardia, Brugada syndrome, Sick sinus syndrome, Wolf Parkinson white syndrome, Heart failure, Cardiomyopathy, valvular heart diseases: aortic stenosis, aortic regurgitation, mitral regurgitation, Tricuspid regurgitation, all other forms of valvular diseases.

**Diabetes mellitus:** Diabetes mellitus includes the condition itself plus its complications such as diabetic foot, diabetic polyneuropathy, diabetic retinopathy, diabetic nephropathy.

**Chronic kidney disease:** Acute tubular necrosis or tubulo interstitial nephritis (TIN), Atypical haemolytic uremic syndrome (aHUS), Amyloidosis, Anti-GBM nephritis, Bartter syndrome, Kidney damage due to medication, Chronic bladder infections/kidney infections, Cryoglobulinemia, Cysts, Cystinosis, Dense deposit disease (DDD), Focal segmental glomerulosclerosis (FSGS), Gitelman syndrome, HNF1 beta associated kidney disease, Horseshoe kidneys, IgA nephropathy, Medullary Sponge Kidneys, Membranous nephropathy, Minimal change disease, mononier Nail-patella syndrome (NPS), Nephrogenic diabetes insipidus, Nephronophthisis, Nephrosclerosis, Nephrotic syndrome, Renal angiomyolipoma's, Kidney filter, Primary hyperoxaluria, Reflux nephropathy, Shrivel kidneys scleroderma, SLE nephritis, Alport's syndrome, Systemic Vasculitis, received dialysis, a kidney transplant, uremia.

**Receiving Immunosuppressive medication:** Azathioprine, Lenalidomide, Methotrexate, Pirfenidone, Epomalidomide, Thalidomide, Abatacept, Apremilast, Baricitinib, Belatacept, Belimumab, Eculizumab, Vedolizumab, Everolimus, Leflunomide, Mycophenolic acid, Sirolimus, Thymocyte globulin, Tofacitinib, Upadacinitib.

**Chronic haematological condition:** Chronic lymphoblastic leukaemia/Acute leukaemia, Thalassemia, Sickle Cell Disease, Haemolytic Disorders, Clotting disorders (Haemophilia, von Willebrand disease, Thrombophilia).

**Chronic neurological condition:** Amyotrophic lateral sclerosis (ALS), Muscular dystrophies (Duchenne, Becker muscular dystrophy), Multiple sclerosis, Parkinson's disease, Guillain barre with still phenomena, Cerebral vascular accidents, (bloody/non-blooded)/transient ischemic attack, Pompeii disease, Dementia.

**Chronic liver disease:** Cirrhosis, Portal hypertension, Chronic hepatitis.

**Received corticosteroids:** Hydrocortisone, Prednisone, Dexamethasone, Methylprednisolone.

**Received antibiotic medications** applies to all forms of antibiotics.

Within the included patients, the main study groups were represented in the sample as follows: 41% for Dutch origin, 9% for African Surinamese origin, 4% for South Asian Surinamese origin, 8% for Moroccan origin, 4% for Turkish origin, 16% for other origins, and 18% for unknown origin (Table 2). The median age was 62 years (IQR, 59–71). There were more males (57%) than females (43%). Median duration of admission was 6 days (IQR, 3–11 days), with 10% re-hospitalised. Dutch origin patients were the oldest (median of 66 years, IQR = 56–75 years). African Surinamese origin patients had the highest prevalence of smoking tobacco (11%), while Dutch origin patients had the highest prevalence of alcohol use disorder (6%). Dutch origin patients also had the highest prevalence of vaccination against COVID-19 before admission (11%). Underlying medical conditions were more prevalent among South Asian Surinamese origin patients (27% had four plus conditions). Turkish origin patients had the highest prevalence of ICU admission (25%). They also received the highest proportion of in-hospital therapies (oxygen, antibiotics, corticosteroids and remdesivir; Table 2).

**Table 2:** Baseline characteristics of patients included in the study by migration background (imputed data).

Categories	Total N = 1886	Dutch origin N = 776	African Surinamese origin N = 177	South Asian Surinamese origin N = 72	Moroccan origin N = 144	Turkish origin N = 67	Other origins N = 310	Unknown origin N = 340	p-value for differences between ethnic groups
**Demographics measures**									
Age (years), median (IQR)	62 (59–71)	66 (56–75)	59 (50–67)	58.5 (50–68)	60 (48–67)	58 (51–68)	57 (47–66)	62 (51–71)	<0.001
Sex, n (%)									
Male	1067 (56.57)	447 (57.60)	69 (38.98)	39 (54.17)	84 (58.33)	37 (55.22)	187 (60.32)	204 (60.00)	<0.001
Female	819 (43.23)	329 (42.40)	108 (61.02)	33 (45.83)	60 (41.67)	30 (44.78)	123 (39.38)	136 (40.00)	
**Behavioural measures**									
Smoking, n (%)									
Current smokers	181 (9.59)	80 (10.31)	20 (11.30)	8 (11.11)	7 (4.86)	5 (7.46)	26 (8.39)	35 (10.29)	<0.001
Former smokers	621 (23.93)	311 (40.08)	58 (32.77)	21 (29.17)	31 (21.53)	28 (41.79)	73 (23.55)	99 (29.12)	
Never smoked	1084 (57.47)	385 (49.61)	99 (55.93)	43 (59.72)	106 (73.61)	34 (50.75)	211 (68.06)	206 (60.59)	
Alcohol use disorder, n (%)									
Yes	87 (4.61)	47 (6.06)	9 (5.08)	3 (4.17)	5 (3.47)	4 (5.97)	7 (2.26)	12 (3.53)	0.089
No	1799 (95.39)	729 (93.94)	168 (94,91)	69 (95.83)	139 (96.52)	63 (94.03)	303 (97.74)	328 (96.47)	
Vaccinated against COVID-19									
Yes	169 (8.96)	86 (11.08)	9 (5.08)	6 (8.33)	11 (7.64)	4 (5.97)	22 (7.10)	31 (9.12)	0.127
No	1717 (91.04)	690 (88.92)	168 (94.92)	66 (91.67)	133 (92.36)	63 (94.03)	288 (92.90)	309 (90.88)	
**Underlying medical conditions**									
Obesity, n (%)									
Yes	530 (28.11)	213 (27.45)	72 (40.67)	17 (23.61)	44 (30.55)	20 (29.85)	85 (27.41)	79 (23.23)	0.003
No	1356 (71.89)	563 (72.55)	105 (59.32)	55 (76.39)	100 (69.44)	47 (70.15)	225 (72.58)	261 (76.76)	
Hypertension, n (%)									
Yes	656 (34.78)	245 (31.57)	94 (53.11)	37 (51.39)	38 (26.39)	30 (44.78)	119 (38.39)	93 (27.35)	<0.001
No	1230 (65.22)	531 (68.43)	83 (46.89)	35 (48.61)	106 (73.61)	37 (55.22)	191 (61.61)	247 (72.65)	
Chronic respiratory condition, n (%)									
Yes	312 (16.54)	146 (18.81)	36 (20.34)	12 (16.67)	15 (10.42)	12 (17.91)	38 (12.26)	53 (15.59)	0.041
No	1574 (83.46)	630 (81.19)	141 (79.66)	60 (83.33)	129 (89.58)	55 (82.09)	272 (87.74)	287 (84.41)	
Chronic cardiovascular condition, n (%)									
Yes	373 (19.78)	183 (23.58)	27 (15.25)	26 (36.11)	35 (24.31)	12 (17.91)	44 (14.19)	46 (13.53)	<0.001
No	1513 (80.22)	593 (76.42)	150 (84.75)	46 (63.89)	109 (75.69)	55 (82.09)	266 (85.81)	294 (86.47)	
Diabetes mellitus, n (%)									
Yes	442 (23.44)	132 (17.01)	56 (31.64)	31 (43.06)	43 (29.86)	20 (29.85)	82 (26.45)	78 (22.94)	<0.001
No	1444 (76.56)	644 (82.99)	121 (68.36)	41 (56.94)	101 (70.14)	47 (70.15)	228 (73.55)	262 (77.06)	
Chronic kidney disease, n (%)									
Yes	186 (9.86)	61 (7.86)	23 (12.99)	22 (30.56)	12 (8.33)	10 (14.93)	37 (11.94)	21 (6.18)	<0.001
No	1700 (90.14)	715 (92.14)	154 (87.01)	50 (69.44)	132 (91.67)	57 (85.07)	273 (88.06)	319 (93.82)	
Receiving immunosuppressive medication, n (%)									
Yes	159 (8.43)	77 (9.92)	17 (9.60)	7 (9.72)	9 (6.25)	6 (8.96)	26 (8.39)	17 (5.00)	0.185
No	1727 (91.57)	699 (90.08)	160 (90.40)	65 (90.28)	135 (93.75)	61 (91.04)	284 (91.61)	323 (95.00)	
Chronic liver disease, n (%)									
Yes	66 (3.50)	22 (2.84)	6 (3.39)	9 (12.50)	2 (1.39)	2 (2.99)	14 (4.52)	11 (3.24)	0.002
No	1820 (96.50)	754 (97.16)	171 (96.61)	63 (87.50)	142 (98.61)	65 (97.01)	296 (95.48)	329 (96.76)	
Malignancy, n (%)									
Yes	100 (5.30)	58 (7.47)	10 (5.65)	2 (2.78)	4 (2.78)	2 (2.99)	8 (2.58)	16 (4.71)	0.016
No	1786 (94.70)	718 (92.53)	167 (94.35)	70 (97.22)	140 (97.22)	65 (97.01)	302 (97.42)	324 (95.29)	
Chronic haematological condition, n (%)									
Yes	82 (4.35)	34 (4.38)	18 (10.17)	1 (1.39)	3 (2.08)	3 (4.48)	12 (3.87)	11 (3.24)	0.004
No	1804 (95.65)	742 (95.62)	159 (89.83)	71 (98.61)	141 (97.92)	64 (95.52)	298 (96.13)	329 (96.76)	
Chronic neurological condition, n (%)									
Yes	190 (10.07)	88 (11.34)	18 (10.17)	9 (12.50)	10 (6.94)	4 (5.97)	32 (10.32)	29 (8.53)	0.474
No	1696 (89.93)	688 (88.66)	159 (89.83)	63 (87.50)	134 (93.06)	63 (94.03)	278 (89.68)	311 (91.47)	
Number of chronic conditions, n (%)									
None	560 (29.69)	219 (28.22)	27 (15.25)	14 (19.44)	53 (36.81)	12 (17.91)	106 (34.19)	129 (37.94)	<0.001
1 chronic condition	467 (24.76)	197 (25.39)	46 (25.98)	15 (20.83)	36 (25.00)	23 (34.33)	72 (23.22)	78 (22.94)	
2 chronic conditions	367 (19.45)	166 (21.39)	37 (20.90)	11 (15.27)	21 (14.58)	11 (16.41)	53 (17.09)	68 (20.00)	
3 chronic conditions	246 (13.04)	105 (13.53)	28 (15.81)	13 (18.05)	16 (11.11)	13 (19.40)	34 (10.96)	37 (10.88)	
4+ chronic conditions	246 (13.04)	89 (11.46)	39 (22.03)	19 (26.39)	18 (12.50)	8 (11.94)	45 (14.51)	28 (8.23)	
**Hospital admission parameters**									
Number of days admitted in hospital, median (IQR)	6.00 (3.00–11.00)	7.00 (4.00–14.00)	4.00 (3.00–9.00)	5.00 (3.00–10.00)	5.00 (3.00–9.25)	7.00 (3.00–10.00)	6.00 (3.50–11.00)	5.00 (3.00–10.00)	<0.001
Rehospitalization, n (%)									
Yes	190 (10.07)	27 (3.48)	5 (2.82)	3 (4.17)	7 (4.86)	4 (5.97)	16 (5.16)	18 (5.29)	0.651
No	1696 (89.93)	749 (96.52)	172 (97.18)	69 (95.83)	137 (95.14)	63 (94.03)	294 (94.84)	322 (94.71)	
Admitted to the ICU, n (%)									
Yes	385 (20.41)	180 (23.20)	24 (13.56)	11 (15.28)	26 (18.06)	17 (25.37)	67 (21.61)	60 (17.65)	0.038
No	1501 (79.59)	596 (76.80)	153 (86.44)	61 (84.72)	118 (81.94)	50 (74.63)	243 (78.39)	280 (82.35)	
Received oxygen, n (%)									
Yes	1608 (85.26)	659 (84.92)	150 (84.75)	56 (77.78)	130 (90.28)	60 (89.55)	263 (84.84)	290 (85.29)	0.022
No	278 (22.32)	117 (15.08)	27 (15.25)	16 (22.22)	14 (9.72)	7 (10.45)	47 (15.16)	50 (14.71)	
Received antibiotics, n (%)									
Yes	861(45.65)	380 (48.97)	72 (40.68)	28 (38.89)	55 (38.19)	35 (52.24)	170 (54.84)	121 (35.59)	<0.001
No	1025 (54.75)	396 (51.03)	105 (59.32)	44 (61.11)	89 (61.81)	32 (47.76)	140 (45.16)	219 (64.41)	
Received corticosteroids, n (%)									
Yes	1091 (57.85)	416 (53.61)	108 (61.02)	44 (61.11)	77 (53.47)	41 (61.19)	182 (58.71)	223 (65.59)	0.011
No	795 (42.15)	360 (46.39)	69 (38.98)	28 (38.89)	67 (46.53)	26 (38.81)	128 (41.29)	117 (34.41)	
Received remdesivir, n (%)									
Yes	181 (9.60)	50 (6.44)	30 (16.95)	7 (9.72)	18 (12.50)	17 (25.37)	34 (10.97)	25 (7.35)	<0.001
No	1705 (90.40)	726 (93.56)	147 (83.05)	65 (90.28)	126 (87.50)	50 (74.63)	276 (89.03)	315 (92.65)	
**Presence of long COVID symptoms**									
Fatigue, n (%)									
Yes	340 (18.03)	127 (16.37)	39 (22.03)	15 (20.83)	29 (20.14)	12 (17.91)	52 (16.77)	66 (19.41)	0.549
No	1546 (81.97)	649 (83.63)	138 (77.97)	57 (79.17)	115 (79.86)	55 (82.09)	258 (83.23)	274 (80.59)	
Dyspnoea, n (%)									
Yes	308 (16.33)	112 (14.43)	32 (18.08)	14 (19.44)	22 (15.28)	16 (23.88)	45 (14.52)	67 (19.71)	0.150
No	1578 (83.67)	664 (85.57)	145 (81.92)	58 (80.56)	122 (84.72)	51 (76.12)	265 (85.48)	273 (80.29)	
Cough, n (%)									
Yes	73 (3.87)	26 (3.35)	8 (4.52)	5 (6.94)	7 (4.86)	6 (8.96)	11 (3.55)	10 (2.94)	0.203
No	1813 (96.13)	750 (96.65)	169 (95.48)	67 (93.06)	137 (95.14)	61 (91.04)	299 (96.45)	330 (97.06)	
Chest pain, n (%)									
Yes	43 (2.28)	17 (2.19)	6 (3.39)	1 (1.39)	7 (4.86)	1 (1.49)	6 (1.94)	5 (1.47)	0.328
No	1843 (97.72)	759 (97.81)	171 (96.61)	71 (98.61)	137 (95.14)	66 (98.51)	304 (98.06)	335 (98.53)	
Heart palpitations, n (%)									
Yes	14 (0.74)	3 (0.39)	1 (0.56)	0 (0.00)	6 (4.17)	0 (0.00)	3 (0.97)	1 (0.29)	<0.001
No	1872 (99.26)	773 (99.61)	176 (99.44)	72 (100.00)	138 (95.83)	67 (100.00)	307 (99.03)	339 (99.71)	
Dizziness, n (%)									
Yes	15 (0.80)	2 (0.26)	2 (1.13)	0 (0.00)	1 (0.69)	3 (4.48)	5 (1.61)	2 (0.59)	0.006
No	1871 (99.20)	774 (99.74)	175 (98.87)	72 (100.00)	143 (99.31)	64 (95.52)	305 (98.39)	338 (99.41)	
Joint/muscle pain, n (%)									
Yes	43 (2.28)	7 (0.90)	6 (3.39)	4 (5.56)	4 (2.78)	6 (8.96)	14 (4.52)	2 (0.59)	<0.001
No	1843 (97.72)	769 (99.10)	171 (96.61)	68 (94.44)	140 (97.22)	61 (91.04)	296 (95.48)	338 (99.41)	
Loss of taste/smell, n (%)									
Yes	32 (1.70)	14 (1.80)	3 (1.69)	0 (0.00)	3 (2.08)	1 (1.49)	7 (2.26)	4 (1.18)	0.859
No	1854 (98.30)	762 (98.20)	174 (98.31)	72 (100.00)	141 (97.92)	66 (98.51)	303 (97.74)	336 (98.82)	
Insomnia, n (%)									
Yes	77 (4.08)	24 (3.09)	10 (5.65)	4 (5.56)	10 (6.94)	4 (5.97)	19 (6.13)	6 (1.76)	0.020
No	1809 (95.92)	752 (96.91)	167 (94.35)	68 (94.44)	134 (93.06)	63 (94.03)	291 (93.87)	334 (98.24)	
Headache, n (%)									
Yes	14 (0.74)	5 (0.64)	1 (0.56)	2 (2.78)	0 (0.00)	0 (0.00)	6 (1.94)	0 (0.00)	0.026
No	1872 (99.26)	771 (99.36)	176 (99.44)	70 (97.22)	144 (100.00)	67 (100.00)	304 (98.06)	340 (100.00)	
Any long COVID symptom, n (%)									
Yes	483 (25.61)	174 (22.42)	59 (33.33)	24 (33.33)	44 (30.56)	24 (35.82)	79 (25.48)	79 (23.24)	0.005
No	1403 (74.39)	602 (77.58)	118 (66.67)	48 (66.67)	100 (69.44)	43 (64.18)	231 (74.52)	261 (76.76)	

**p-value for differences between ethnic groups** considered statistically significant at values less than 0.05.

**Alcohol use disorder** based on Diagnostic and Statistical Manual of Mental Disorders (DSM–5) criteria.

**Vaccinated against COVID-19** refers to receipt of any time of COVID-19 before admission irrespective of number of doses at admission.

**Obesity:** body mass index ≥30 kg/m^2^, data collected as a yes or no in the electronic medical records.

**Chronic respiratory condition:** Asthma, Alpha 1 trypsin deficiency, Asbestosis, Cryptogenic organizing pneumonia (COP), LAM, LICS, Broncho-pulmonary dysplasia or Primary ciliary dyskinesia, Bronchiectasis, Cystic fibrosis, COPD (chronic bronchitis, emphysema), Lung fibrosis, Sarcoidosis, Obstructive sleep apnoea, Pulmonary hypertension.

**Chronic cardiovascular condition:** Myocardial infarction, Cardiac arrhythmias: AVNRT, Atrial fibrillation, (supra)ventricular tachycardia, Ventricular tachycardia, Brugada syndrome, Sick sinus syndrome, Wolf Parkinson white syndrome, Heart failure, Cardiomyopathy, valvular heart diseases: aortic stenosis, aortic regurgitation, mitral regurgitation, Tricuspid regurgitation, all other forms of valvular diseases.

**Chronic kidney disease:** Acute tubular necrosis or tubulo interstitial nephritis (TIN), Atypical haemolytic uremic syndrome (aHUS), Amyloidosis, Anti-GBM nephritis, Bartter syndrome, Kidney damage due to medication, Chronic bladder infections/kidney infections, Cryoglobulinemia, Cysts, Cystinosis, Dense deposit disease (DDD), Focal segmental glomerulosclerosis (FSGS), Gitelman syndrome, HNF1 beta associated kidney disease, Horseshoe kidneys, IgA nephropathy, Medullary Sponge Kidneys, Membranous nephropathy, Minimal change disease, mononier Nail-patella syndrome (NPS), Nephrogenic diabetes insipidus, Nephronophthisis, Nephrosclerosis, Nephrotic syndrome, Renal angiomyolipoma's, Kidney filter, Primary hyperoxaluria, Reflux nephropathy, Shrivel kidneys scleroderma, SLE nephritis, Alport's syndrome, Systemic Vasculitis.

**Receiving Immunosuppressive medication:** Azathioprine, Lenalidomide, Methotrexate, Pirfenidone, Epomalidomide, Thalidomide, Abatacept, Apremilast, Baricitinib, Belatacept, Belimumab, Eculizumab, Vedolizumab, Everolimus, Leflunomide, Mycophenolic acid, Sirolimus, Thymocyte globulin, Tofacitinib, Upadacinitib.

**Diabetes mellitus** includes its complications such as diabetic foot, diabetic polyneuropathy, diabetic retinopathy, diabetic nephropathy.

**Chronic haematological condition:** Chronic lymphoblastic leukaemia/Acute leukaemia, Thalassemia, Sickle Cell Disease, Haemolytic Disorders, Clotting disorders (Haemophilia, von Willebrand disease, Thrombophilia).

**Chronic neurological condition:** Amyotrophic lateral sclerosis (ALS), Muscular dystrophies (Duchenne, Becker muscular dystrophy), Multiple sclerosis, Parkinson's disease, Guillain barre with still phenomena, Cerebral vascular accidents, (bloody/non-blooded)/transient ischemic attack, Pompeii disease, Dementia.

**Chronic liver disease:** Cirrhosis, Portal hypertension, Chronic hepatitis.

**Received corticosteroids:** Hydrocortisone, Prednisone, Dexamethasone, Methylprednisolone.

**Received antibiotic medications** applies to all forms of antibiotics.

### Nature of long COVID symptoms

**Fig. 2:**
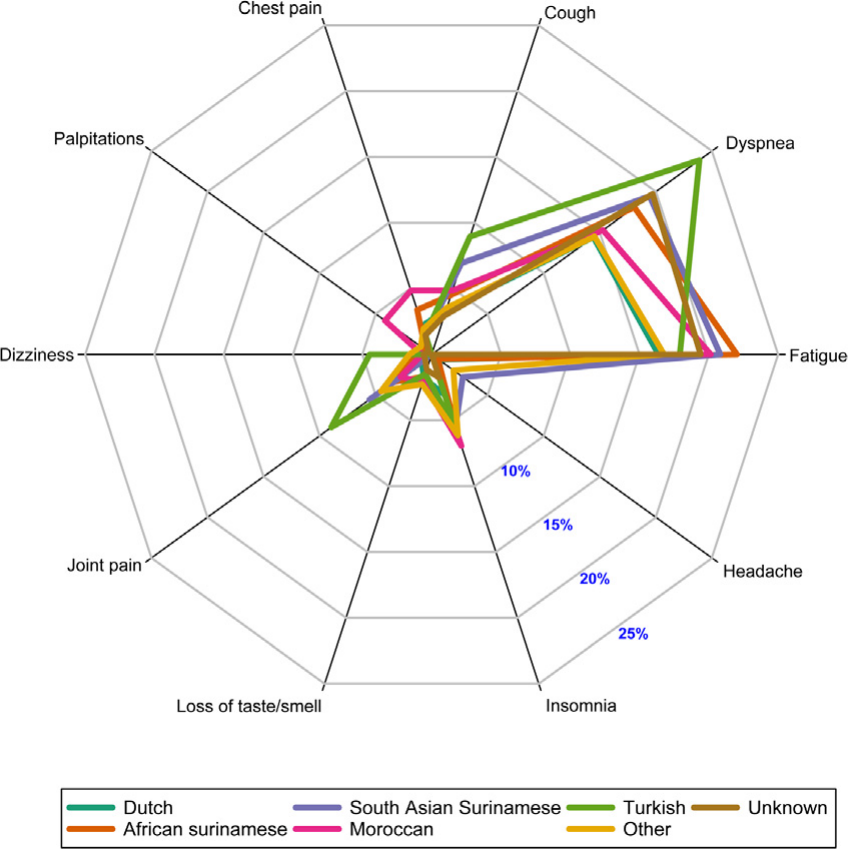
**Nature of long COVID symptoms (imputed data).** Radar plots depict the proportion of each symptom per migration background, and the symptom patterns per migration background.

The most common symptoms reported at 12 weeks post-hospital discharge were dyspnoea (18%) and fatigue (16%; Table 2; Fig. 2). Reports of dizziness, joint and muscle pain were highest among Turkish origin patients (p for trend < 0.001). On the other hand, reports of heart palpitations, and insomnia were highest among Moroccan origin patients (p for trend < 0.002). Reports of headache were highest among South Asian Surinamese (p for trend = 0.026). There were no statistically different proportions in reports of fatigue, dyspnoea, cough, chest pain and loss of taste/smell by migration background.

### Age and sex adjusted incidence proportions of long COVID

**Fig. 3:**
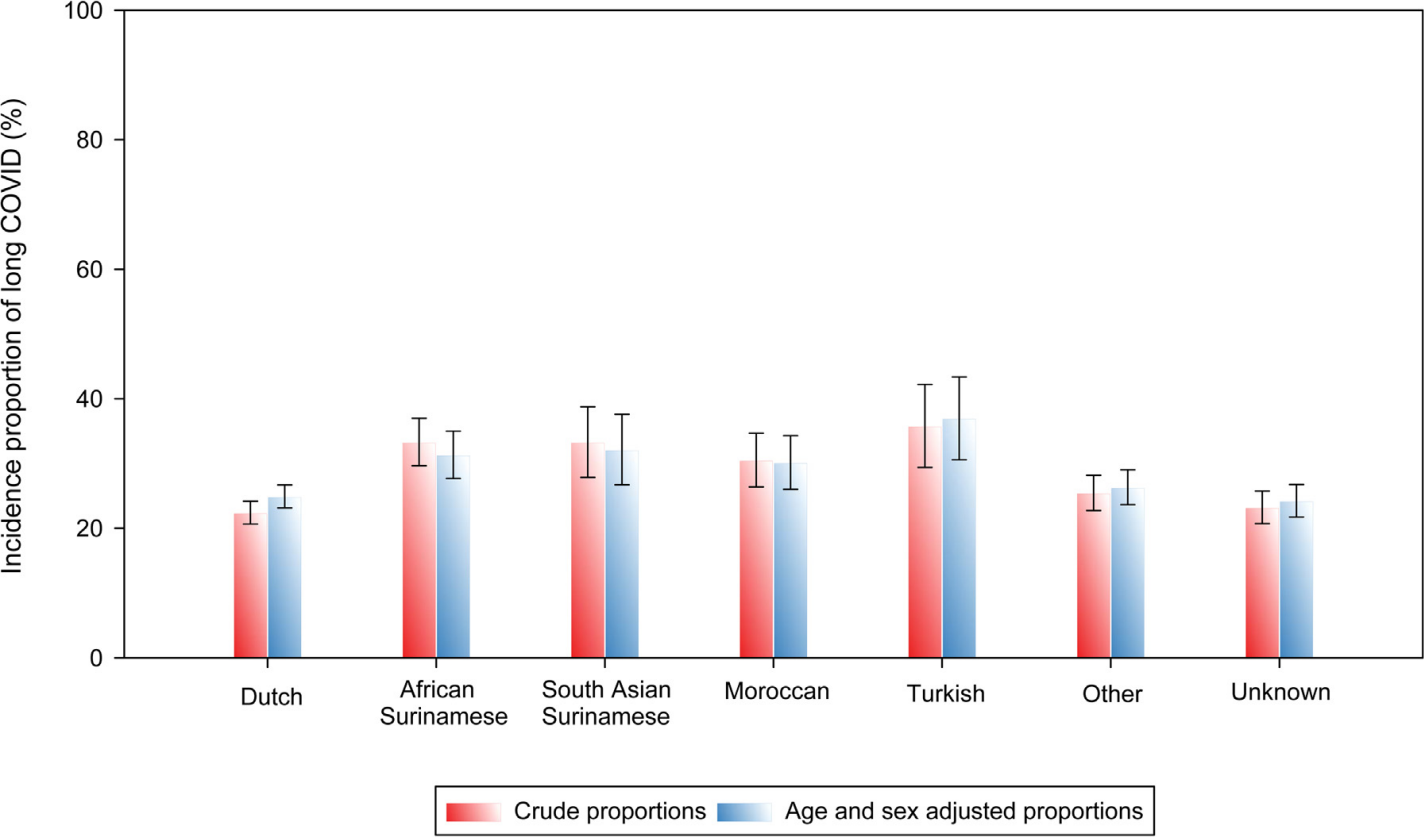
**Incidence proportions of long COVID (imputed data).** Bar graph depicts the incidence proportions of long COVID per migration background.

A total of 483 patients (26%, 95% CI 24–28%) had any ongoing symptoms at 12 weeks post-hospital discharge. Age- and sex-adjusted incidence proportions of long COVID (i.e., any of the persistent symptoms) were higher among Turkish origin patients (37%, 95% CI 31–43%), African Asian Surinamese patients (31%, 95% CI 28–35%), South Asian Surinamese patients (32%, 95% CI 27–38%), Moroccan patients (31%, 95% CI 27–34%) than the Dutch origin patients (25%; 95% CI 23–26%, Fig. 3).

### Clinical predictors of long COVID

After one-step robust Poisson regressions with candidate clinical predictors in the total population (Table 3), the following factors were positively associated with presence of any long COVID symptom in our model: female sex (IRR = 1.26, 95% CI 1.08–1.48), duration of hospital admission (IRR = 1.01, 95% CI 1.01–1.02), admission to the ICU (IRR = 1.41, 95% CI 1.13–2.20) and receiving oxygen (IRR = 1.70, 95% CI 1.32–2.20). On the other hand, receiving corticosteroids during hospitalisation was negatively associated with any long COVID symptom in our model (IRR = 0.64, 95% CI 0.54–0.76). There were no statistically significant interaction effects between severity of COVID-19 (admission to ICU and receiving oxygen) with receiving corticosteroid therapy. Despite being important, vaccination status against COVID-19 was not identified as a clinical predictor of long COVID in our model. Additionally, among those admitted to ICU, duration of invasive ventilation was positively associated with presence of any long COVID symptom (IRR = 1.007, 95% CI 1.000–1.019, Appendix 3).

**Table 3:** Clinical predictors of long COVID (imputed data).

Variable	Categories	Variance inflation factor (VIF)	Model 1 PR (95% CI)
Migration background			
	Dutch origin		Reference
	African Surinamese origin		**1.40(1.09–1.81)**
	South Asian Surinamese origin		**1.53 (1.07–2.19)**
	Moroccan origin		**1.34 (1.01–1.77)**
	Turkish origin		**1.44 (1.03–2.00)**
	Other origin		1.08 (0.85–1.37)
	Unknown origin	1.05	1.13 (0.88–1.43)
Age (years)		1.18	0.98 (0.99–1.02)
Sex	Male		Reference
	Female	1.05	**1.26 (1.08–1.48)**
Smoking	Never smoked		Reference
	Current smoker		1.06 (0.89–1.25)
	Past smoker	1.03	0.96 (0.73–1.27)
Alcohol consumption	No		Reference
	Yes	1.02	0.67 (0.42–1.06)
Vaccinated against COVID-19	No		Reference
	Yes	1.05	0.75 (0.54–1.08)
Number of chronic health conditions	None		Reference
	1 condition		0.92 (0.75–1.14)
	2 conditions		0.85 (0.67–1.09)
	3 conditions		1.02 (0.79–1.31)
	4+ conditions	1.18	0.99 (0.76–1.29)
Number of days admitted in hospital		1.03	**1.01 (1.01–1.02)**
Rehospitalization	No		Reference
	Yes	1.50	1.07 (0.75–1.53)
Admitted to the ICU	No		Reference
	Yes	1.78	**1.41 (1.13–1.74)**
Received oxygen	No		Reference
	Yes	1.49	**1.70 (1.32–2.20)**
Received antibiotics	No		Reference
	Yes	1.52	1.01 (0.84–1.22)
Received corticosteroids	No		Reference
	Yes	1.37	**0.64 (0.54–0.76)**
Received remdesivir	No		Reference
	Yes	1.09	1.23 (0.97–1.57)

**PR** = prevalence ratios with 95% confidence interval obtained via robust Poisson regression models.

**Variance inflation factor** (VIF) analysis to identify predictors that are correlated. VIF scores greater than five indicate high correlation between predictors.

**Model 1**: multivariate robust Poisson regressions.

**Vaccinated against COVID-19** refers to receipt of any time of COVID-19 before admission irrespective of number of doses at admission.

**Received corticosteroids:** hydrocortisone, prednisone, dexamethasone, methyldprednisone.

**Received antibiotic medications** applies to all forms of antibiotics.

Bold values inside the table signify statistically significant findings.

### Associations between migration background and long COVID

Compared to the Dutch origin patients, patients with African Surinamese, South Asian Surinamese, Moroccan and Turkish origin had a higher risk of reporting any long COVID symptom than Dutch origin patients after adjustment for age, sex, smoking, vaccination status against COVID-19, number of co-morbidities, duration of hospital admission, admission to ICU, receiving oxygen, corticosteroids, and remdesivir therapy (IRRs = 1.41, 95% CI 1.10–1.82; 1.54, 95% CI 1.07–2.11, 1.39, 95% CI 1.05–1.83 and 1.45, 95% CI 1.04–2.02 respectively; Table 4). With respect to individual long COVID symptoms, patients with Turkish origin had higher risk of reporting a cough after adjustments for all the relevant variables when compared to Dutch origin patients (IRR = 2.33, 95% CI 1.01–5.49).

**Table 4:** Association of migration background with incidence of long COVID and individual long COVID symptoms (imputed data).

Long COVID symptoms	(Long COVID per group total)	Crude model	Fully adjusted model
	N	PR (95% CI)	PR (95% CI)
**General incidence of long COVID**			
Dutch origin	174/776	1.00 (ref)	1.00 (ref)
African Surinamese origin	59/177	1.49 (1.16–1.90)	**1.41 (1.10–1.82)**
South Asian Surinamese origin	24/72	1.49 (1.05–2.11)	**1.54 (1.07–2.21)**
Moroccan origin	44/144	1.36 (1.03–1.80)	**1.39 (1.05–1.83)**
Turkish origin	24/67	1.60 (1.13–2.26)	**1.45 (1.04–2.02)**
Other origin	79/310	1.14 (0.90–1.43)	1.10 (0.87–1.39)
Unknown origin	79/340	1.04 (0.82–1.31)	1.14 (0.90–1.44)
**Fatigue**			
Dutch origin	127/776	1.00 (ref)	1.00 (ref)
African Surinamese origin	39/177	1.35 (0.98–1.85)	1.21 (0.87–1.69)
South Asian Surinamese origin	15/72	1.27 (0.79–2.05)	1.25 (0.77–2.03)
Moroccan origin	29/144	1.23 (0.86–1.77)	1.15 (0.79–1.66)
Turkish origin	12/67	1.09 (0.64–1.87)	1.06 (0.62–1.81)
Other origin	52/310	1.02 (0.76–1.38)	0.94 (0.57–1.65)
Unknown origin	66/340	1.19 (0.91–1.55)	1.25 (0.95–1.64)
**Dyspnoea**			
Dutch origin	112/776	1.00 (ref)	1.00 (ref)
African Surinamese origin	32/177	1.25 (0.88–1.79)	1.25 (0.86–1.81)
South Asian Surinamese origin	14/72	1.35 (0.82–2.22)	1.46 (0.87–2.42)
Moroccan origin	22/144	1.06 (0.69–1.61)	1.11 (0.72–1.45)
Turkish origin	16/67	1.65 (1.04–2.62)	1.56 (0.99–2.47)
Other origin	45/310	1.01 (0.73–1.38)	1.04 (0.75–1.45)
Unknown origin	67/340	1.37 (1.04–1.80)	**1.51 (1.14–1.99)**
**Cough**			
Dutch origin	26/776	1.00 (ref)	1.00 (ref)
African Surinamese origin	8/177	1.35 (0.62–2.93)	1.26 (0.55–2.88)
South Asian Surinamese origin	5/72	2.07 (0.82–5.23)	2.02 (0.78–5.25)
Moroccan origin	7/144	1.45 (0.64–3.28)	1.29 (0.56–2.99)
Turkish origin	6/67	2.67 (1.14–6.27)	**2.33 (1.01–5.49)**
Other origin	11/310	1.06 (0.53–2.12)	0.92 (0.45–1.89)
Unknown origin	10/340	0.88 (0.43–1.80)	0.85 (0.41–1.77)
**Chest pain**			
Dutch origin	17/776	1.00 (ref)	1.00 (ref)
African Surinamese origin	6/177	1.55 (0.62–3.87)	1.35 (0.48–3.76)
South Asian Surinamese origin	1/72	0.63 (0.09–4.70)	0.63 (0.08–5.05)
Moroccan origin	7/144	2.22 (0.94–5.25)	1.83 (0.78–4.31)
Turkish origin	1/67	0.68 (0.09–5.04)	0.62 (0.09–4.24)
Other origin	6/310	0.88 (0.35–2.22)	0.74 (0.28–1.90)
Unknown origin	5/340	0.67 (0.25–1.80)	0.68 (0.24–1.93)
**Insomnia**			
Dutch origin	24/776	1.00 (ref)	1.00 (ref)
African Surinamese origin	10/177	1.83 (0.89–3.75)	1.88 (0.87–4.06)
South Asian Surinamese origin	4/72	1.80 (0.64–5.03)	2.28 (0.82–6.33)
Moroccan origin	10/144	2.25 (1.10–4.59)	2.37 (1.15–4.86)
Turkish origin	4/67	1.93 (0.69–5.40)	1.92 (0.69–5.33)
Other origin	19/310	1.98 (1.10–3.57)	**2.07 (1.10–3.89)**
Unknown origin	6/340	0.57 (0.24–1.38)	0.73 (0.30–1.77)

The migration background groups with one- or no-person reporting symptoms of heart palpitations, dizziness, joint and muscular discomfort, and loss of taste and smell were excluded from this table.

**PR** = Prevalence ratios obtained via robust Poisson regression and their 95% confidence intervals. Long COVID/symptoms as outcomes, migration background as the predictor.

**Fully adjusted model**: adjusted for age + sex + statistically significant determinants of long COVID (i.e., number of days admitted to hospital + admission to ICU + receiving oxygen therapy + receiving steroid therapy) + other non-statistically significant determinants of long COVID (i.e., smoking + vaccination status against COVID-19 + number of comorbidities + receiving remdesivir therapy).

Interactions between oxygen therapy and steroid therapy, as well as between admission to ICU and steroid therapy were not statistically significant hence not included as additional effects in the models.

Bold values inside the table signify statistically significant findings.

### Persistence of long COVID at 1 year

**Fig. 4:**
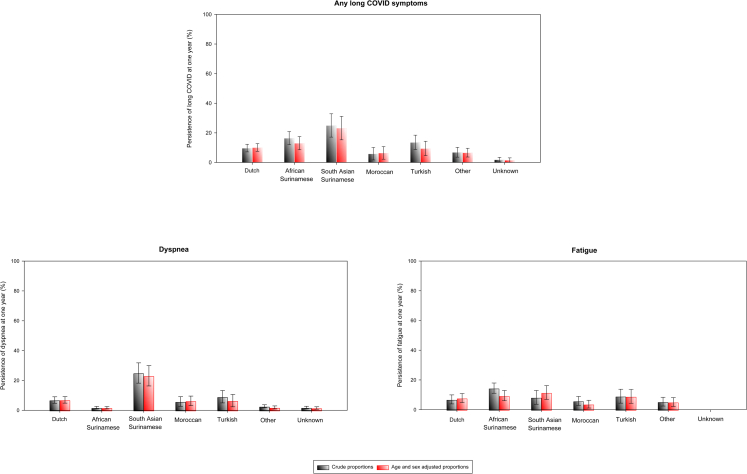
**Incidence proportions of long COVID persistence (imputed data).** Bar graph depicts the incidence proportions of long COVID at one year among those with long COVID at three months, per migration background. ***Persistence of any long COVID symptoms*** among patients of Dutch origin = 14/122 (13%), African Surinamese origin = 9/33 (27%), South Asian Surinamese origin = 6/17 (35%), Moroccan origin = 2/22 (9%), Turkish origin = 3/19 (16%), Other origin = 5/37 (14%), Unknown Origin = 1/45 (2%). ***Persistence of dyspnoea*** among patients of Dutch origin = 10/122 (9%), African Surinamese origin = 1/33 (3%), South Asian Surinamese origin = 6/17 (35%), Moroccan origin = 2/22 (9%), Turkish origin = 2/19 (11%), Other origin = 2/37 (5%), Unknown Origin = 1/45 (2%). ***Persistence of fatigue*** among patients of Dutch origin = 10/122 (9%), African Surinamese origin = 8/33 (24%), South Asian Surinamese origin = 2/17 (12%), Moroccan origin = 2/22 (9%), Turkish origin = 2/19 (11%), Other origin = 3/37 (9%), Unknown Origin = 0/45 (0%).

Out of the 483 patients with long COVID at 12 weeks post-hospital discharge, 404 (84%) had a follow up of up to one year. From this group, 119 (29%) did not return to the clinic for appointment. The proportions of loss to follow up ranged from 21% in Turkish origin patients to 50% in Moroccan origin patients (p for trend <0.001; Appendix 4). Dutch origin patients had an intermediate loss to follow up of 36%. A total 285 (71%) reported whether long COVID symptoms persisted or not (i.e., by either returning for follow-up at the post-COVID clinic or reporting to be feeling well/free from any long COVID symptoms from home). A total of 40 (14%, 95% CI 10–19%) patients reported persistence of long COVID symptoms. Dyspnoea (8%, 95% CI 5–11%) and fatigue (9%, 95% CI 6–13%) were still the most common symptoms (Fig. 4). Age and sex adjusted incidence proportions of long COVID persistence were higher among South Asian Surinamese (36%, 95% 28–42%) and African Surinamese (21%, 95% 17–28%) than the Dutch origin patients (13%, 95% CI 10–17%; Fig. 4).

### Sensitivity analyses

In one-stepwise robust Poisson regression models repeated with unimputed data, the clinical predictors of long COVID were still found to be female sex, duration of hospital admission, admission to the ICU, receiving oxygen, and corticosteroids during hospitalisation (Appendix 5). Furthermore, robust Poisson regression models with unimputed data showed that patients with African Surinamese, South Asian Surinamese, Moroccan and Turkish origin had a higher risk of long COVID than Dutch origin patients after adjustments for age, sex, smoking, vaccination status against COVID-19, number of co-morbidities, duration of hospital admission, admission to ICU, receiving oxygen, cortico steroids, and remdesivir therapy (Appendix 6). Lastly, all multiple imputation datasets showed that patients with African Surinamese, South Asian Surinamese, Moroccan and Turkish origin had a higher risk of long COVID than Dutch origin patients after adjustment for all the relevant variables (Appendix 7).

## Discussion

### Key findings

In our study on differences in incidence, nature of symptoms, clinical predictors, and duration of long COVID by migration background among individuals hospitalised for COVID-19 in the Netherlands, we found that about one quarter of patients had at least one long COVID symptom. Age- and sex-adjusted long COVID incidence proportions were highest in Surinamese, Turkish and Moroccan origin patients. Symptoms such as dizziness, joint and muscle pain, heart palpitations, insomnia, and headache varied by migration background. Clinical Predictors of long COVID were female sex, duration of hospital admission, admission to ICU, and receiving oxygen or corticosteroid therapy during hospitalisation. African Surinamese, South Asian Surinamese, Moroccan and Turkish origin Patients had a higher risk of long COVID than Dutch origin patients. Only 14% of any long COVID symptoms were still persistent at one-year post-discharge mainly among the South Asian Surinamese origin patients.

### Discussion of key findings

The long COVID incidence proportion of 26% between January and December 2021 in our study is close to the estimate of 21% from another large perspective, population-based Dutch study in non-hospitalised individuals conducted between March 2020, and August 2021 (when only core long COVID symptoms were considered).^18^ On the other hand, our incidence proportion is lower than other estimates from the Netherlands and the world at large. For instance, another cohort study of hospitalised and non-hospitalised individuals conducted between May 2020 and May 2021 in the Netherlands, and included more symptoms, reported an overall incidence proportion of 60%.^19^ Furthermore, a meta-analysis of 33 studies in hospitalised and non-hospitalised individuals had overall long COVID prevalence of up to 50% (54% for hospitalised, and 34% for non-hospitalised individuals).^1^ Additionally, long COVID prevalence for the European region was at 44%.^1^ This wide variation in long COVID incidence across cohorts could possibly arise from the differences in the study population (e.g., hospitalised vs non-hospitalised), time point in the Coronavirus pandemic, as well as type and number of symptoms assessed in each cohort.

The higher risk of long COVID among African Surinamese, South Asian Surinamese, Turkish and Moroccan origin patients than the Dutch origin patients was expected. This is based on the finding that these patient groups experienced higher COVID-19 morbidity and mortality than the Dutch origin patients, which shows that these groups had persons more vulnerable to COVID-19 and likely its long term implications.^6^^,^^8^ Our findings are in line with another population based study in the UK that shows that ethnic minority groups (black and mixed population) reported longer COVID symptoms than the white participants.^11^ On the contrary, our findings differ to those reported in another longitudinal study in the UK of a combined sample of hospitalised and non-hospitalised adults where South Asian and black populations had lower odds of long COVID relative to the white population.^12^

In our cohort, long COVID incidence was positively associated with female sex, duration of hospital admission, admission to the ICU, and receiving oxygen during hospitalisation, but also negatively with receiving corticosteroids during hospitalisation. While sex is mostly considered as a confounder, duration of hospital admission, admission to the ICU, and receiving oxygen and corticosteroids during hospitalisation could be considered as explanatory factors for the higher risk of long COVID among patients with a migration background. First, it could be that patients who stayed longer in the hospital, received oxygen, and were admitted to the ICU, had severe COVID-19 (more organ damage) than those who did not thereby increasing the risk of long COVID. Second, corticosteroids are potent anti-inflammatory and immunosuppressive agents. Treatment with corticosteroids reduces multi-system inflammation (hallmark of COVID-19), which could also potentially reduce the long COVID risk.^20^ The higher risk of long COVID among African Surinamese, South Asian Surinamese, Moroccan and Turkish origin patients than Dutch origin patients was still apparent after adjusting for duration of hospital admission ICU admission, and receiving oxygen and corticosteroid therapies, as well as other biologically relevant clinical factors such as vaccination status against COVID-19, smoking, presence of co-morbidities and treatment with remdesivir did not alter the findings. This seems to suggest clearly that other unmeasured factors such as social factors (e.g., socio-economic status, duration of stay in the Netherlands and acculturation levels), inflammatory markers during hospitalisation, and health behaviours after hospital discharge (e.g., consumption of healthy diets and nutritional supplements), and genetic factors, could have possibly further explained our findings but were not available for inclusion in our study.

As reported in many previous studies, dyspnoea and fatigue were also the most common symptoms in our study.^2^ However, our study is the first to report on how the individual long COVID symptoms vary by migration background. For example, reports of dizziness, joint and muscle pain were highest among Turkish origin patients. On the other hand, reports of heart palpitations, and insomnia were highest among Moroccan origin patients. Previous studies have shown that COVID-19 symptoms vary in different immune contexts.^21^ Moreover, ethnicity and migration background are key determinants of immunological responses.^22^ Variation in the nature of long COVID symptoms by migration background could therefore relate to the interface between SARS-CoV-2 characteristics (i.e., variants of concern), host genotype and immunological responses.

The 86% resolution rate of long COVID symptoms at 1-year (i.e., 14% persistence of long COVID symptoms) in our study falls in line with resolution rates from previous studies.^23^, ^24^, ^25^, ^26^ Broad rates of symptom resolution have been reported across studies ranging from 22% among individuals who developed acute respiratory distress syndrome (ARDS) during hospitalisation to 86% among general hospitalised patients.^26^ In our study, we observed differences in symptoms resolution by migration background at 1 year post-hospital discharge. Specifically, South Asian and African Surinamese origin patients had lower rates of symptom resolution than the Dutch origin patients. On the other hand, the symptom resolution among Moroccan origin patients appeared to be higher than that of Dutch origin patients. While populations with a migration background generally exhibit a higher risk for COVID, this observation is in line with a study in Spain that showed lower COVID-19 risk among Moroccan origin than the Spanish origin population.^27^ Clinical predictors of symptom resolution at one-year could not be evaluated due to small sample sizes, further larger studies should explore how various treatments and health behaviours after a long COVID diagnosis influence symptom resolution in the long term across ethnic groups.

Our research has two major implications. First, our findings call for additional research to assess the degree of functional limitation among long COVID patients, which can aid in the planning of appropriate healthcare interventions and the earliest possible return to normal life (including earning a living). Second, our findings call for additional research to determine whether patients with a migration background have less access to post COVID health care, which can aid in the planning of accessible healthcare interventions.

### Strengths and limitations

The biggest strength of our study is that it consists of a multi-ethnic cohort, which enabled an in-depth assessment of long COVID by migration background. Second, the longitudinal prospective nature of our electronic data collection minimised recall bias. Third, the study utilises electronic medical records from the hospital, which provided accurate, and up-to-date information about hospitalised individuals at the point of care. However, there are several limitations. First, because we used electronic records retrospectively, we did not have data on socio-economic status, immunological markers, and health behaviours after hospital discharge, which would have enriched our analyses. Second, the sample size included in our study was only 63% of the original sample, which could lead to selection bias. Infact, patients that were not included in this study were likely to be older, to be male, to be smokers, to consume more alcohol, to be more vaccinated, to be less obese, to have low levels of hypertension, to have more chronic kidney disease, to have more malignancies, to receive less corticosteroids, to be hospitalised longer, to be admitted to the ICU, to receive more oxygen, corticosteroids and remdesivir therapies than the patients included. As a result, the findings may not be completely generalizable to all multi-ethnic COVID-19 patients hospitalized in Amsterdam. Third, it is possible that symptoms reported at 12 weeks were not related to COVID-19 but to post-intensive care syndrome (PICS) or other clinical entities with similar signs and symptoms but different underlying pathology.^28^ However, differences in long COVID by migration background were still prominent after adjusting for admission to ICU, which shows that our findings were not attributable to admission to ICU, although the role of other clinical conditions that mimic long COVID could not be excluded.^28^ Moreover, our long COVID rate (26%) is close to the 20% national estimate by the (National Public Health Institute; RIVM) in the general population, which increases the credibility of the findings.^29^ Fourth, there was a large group of patients with unknown origin in our study (18%). We do not know how allocation of these patients to their correct ethnic origins would have influenced our findings. Fifth, our sample size at 1 year was too limited to examine the clinical predictors of symptom persistence across ethnic groups after a diagnosis of long COVID (at 3 months). Our sample also limited our ability to assess the clinical predictors of long COVID by migration background. Sixth, we only studied patients with first generation migration background, the findings cannot be extrapolated to patients with a second-generation migration background. Moreover, it is also possible that some patients of second-generation background were classified with into Dutch origin group. However, hospitalisation for COVID-19 is more common among the first generation (older) that second generation (younger), hence the proportion of misclassified second generation migrants is likely to be small and less influential on the findings. Seventh, some patient groups were more likely to be lost to follow up (e.g., Moroccan origin) in comparison to the others (e.g., Turkish origin). This might have led to selection bias at follow-up especially among those with challenges in accessing health care. Eighth, missing values were imputed. Repeated statistical analyses with un-imputed data produced results like those obtained with imputed data, increasing the validity our findings. Ninth, symptom reporting is subjective. Although differences in disease symptoms by migration background has not been well characterised in the Netherlands, the relative frequency of long COVID symptoms reported by different patient groups may be related to cultural or linguistic background. However, the decrease in the number of symptoms reported at 1 year seems to suggest that this bias might be small. Tenth, there were statistically significant differences in the loss to follow up at 1-year by migration background. This could have contributed to selection bias in the assessment of persistence of long COVID at the 1-year time point. Lastly, we could not exhaust all the symptoms of long COVID, which might have resulted in underestimation of long COVID at 12 weeks post-hospital discharge and an overestimation of resolution of symptoms in this group at 12 months. However, the symptoms assessed in our study are the most commonly reported symptoms per NICE guidelines.^5^

## Conclusion

In a multi-ethnic cohort of COVID-19 hospitalized individuals, one fourth of patients report ongoing symptoms after a COVID-19 admission, some of which vary by migration background. African Surinamese, South Asian Surinamese and Turkish origin patients have higher risk of long COVID than Dutch origin patients. Long COVID risk in the total population is related to female sex, duration of hospital admission, ICU admission, receiving oxygen and corticosteroid therapies during hospitalisation. Majority of long COVID symptoms disappear within a year of hospital discharge in most individuals. Studies assessing the differences in access to post-COVID health care, as well as the spectrum of functional limitation from long COVID are needed to help plan appropriate for and accessible healthcare interventions.

## Contributors

F.P.C., B.A., M.R., M.N., and C.A. conceived and designed the study. F.P.C. analysed the data. B.A. cross-checked and verified the analyses. F.P.C. wrote the paper with B.A. and C.A. All authors F.P.C., B.A., M.v.V., K.K., P.S., J.v.E., W.J.W., M.R., M.P., K.S., M.N., and C.A. participated in drafting the article or revising it critically for content.

## Data sharing statement

Data used for the study is available upon request.

## Declaration of interests

B.A. and M.v.V. report Patient Led Research Collaboration (PLRC) grant paid to the host institution. W.J.W. reports consultancy fees from Pfizer, GSK, and AstraZeneca paid to the host institution, as well as being a member of GSK data and safety monitoring board (fees paid to host institution). All other authors declare that they have no competing interests.

## Appendix A Supplementary data

Appendices 1–7
