# Supplementary material for: Differences in incidence, nature of symptoms, and duration of long COVID among hospitalised migrant and non-migrant patients in the Netherlands: a retrospective cohort study
Source: Lancet Reg Health Eur. 2023 Apr 7;29:100630. doi: 10.1016/j.lanepe.2023.100630 (PMC10079482; doi:10.1016/j.lanepe.2023.100630)
Supplement: Appendices 1–7 [file mmc1.pdf]

# SUPPLEMENTARY MATERIALS

## APPENDIX 1. List of medical conditions and drug treatments

**Alcohol use disorder**

Based on Diagnostic and Statistical Manual of Mental Disorders (DSM–5) criteria.

**Obesity**

Body mass index $\geq 30$ kg/m$^2$, data collected as a yes or no in the electronic medical records

**Chronic respiratory condition**

Asthma, Alpha 1 trypsin deficiency, Asbestosis, Cryptogenic organizing pneumonia (COP), Lymphangioleiomyomatosis (LAM), Autosomal recessive lung disease immuno-deficiency and chromosome breakage syndrome (LICS), Broncho-pulmonary dysplasia or primary ciliary dyskinesia, Bronchiectasis, Cystic fibrosis, COPD (chronic bronchitis, emphysema), lung fibrosis, Sarcoidosis, Obstructive sleep apnoea, Pulmonary hypertension.

**Chronic cardiovascular condition**

Myocardial infarction, Cardiac arrhythmias: AVNRT, Atrial fibrillation, Supraventricular tachycardia, Ventricular tachycardia, Brugada syndrome, Sick sinus syndrome, Wolf Parkinson white syndrome, Heart failure, Cardiomyopathy, Valve disease: Aortic valve stenosis, Aortic valve insufficiency, Mitral regurgitation, Tricuspid regurgitation, all other valvular heart diseases.

**Diabetes Mellitus**

Diabetes mellitus includes the condition itself plus its complications such as diabetic foot, diabetic polyneuropathy, diabetic retinopathy, diabetic nephropathy.

**Chronic kidney disease**

Acute tubular necrosis or tubulo interstitial nephritis (TIN), Atypical haemolytic uremic syndrome (aHUS), Amyloidosis, Anti-GBM nephritis, Bartter syndrome, Kidney damage due to medication, Chronic bladder infections/kidney infections, Cryoglobulinemia, Cysts, Cystinosis, Dense deposit disease (DDD), Focal segmental glomerulosclerosis (FSGS), Gitelman syndrome, HNF1 beta associated kidney disease, Horseshoe kidneys, IgA nephropathy, Medullary Sponge Kidneys, Membranous nephropathy, Minimal change disease, Mononie, Nail-patella syndrome (NPS), Nephrogenic diabetes insipidus, Nephronophthisis, Nephrosclerosis, Nephrotic syndrome, Renal angiomyolipoma's, Kidney filter, Primary hyperoxaluria, Reflux nephropathy, Shrivel kidneys scleroderma, SLE nephritis, Alport's syndrome, Systemic Vasculitis, received dialysis, a kidney transplant, uremia.

**Receiving Immunosuppressive medication**

Azathioprine, Lenalidomide, Methotrexate, Pirfenidone, Epomalidomide, Thalidomide, Abatacept, Apremilast, Baricitinib, Belatacept, Belimumab, Eculizumab, Vedolizumab, Everolimus, Leflunomide, Mycophenolic acid, Sirolimus, Thymocyte globulin, Tofacitinib, Upadacinitib, Prednisolone if used for a longer period (>3 weeks) and doses higher than 5 mg per day on the day of admission.

**Chronic haematological condition**

Chronic lymphoblastic leukaemia/Acute leukaemia, Thalassemia, Sickle Cell Disease, Haemolytic Disorders, Clotting disorders (haemophilia, von Willebrand disease, thrombophilia).

**Chronic neurological condition**

Amyotrophic lateral sclerosis (ALS), Muscular dystrophies (Duchenne, Becker muscular dystrophy), Multiple sclerosis, Parkinson's disease, Guillain barre with still phenomena, Cerebral vascular accidents, (bloody/non-blooded) /transient ischemic attack, Pompeii disease, Dementia.

**Chronic liver disease**

Cirrhosis, Portal hypertension, Chronic hepatitis.

**Received corticosteroids**

Hydrocortisone, Prednisone, Dexamethasone, Methylprednisolone.

**Received antibiotic medications**

Applies to all forms of antibiotics

**Received antivirals:**

remdesivir

**Appendix 2.** Proportions of missing values that were imputed for each variable.

| **Categories** | **Number of missing values (Out of N=1886)** | **Percentage missing (%)** | **Imputed (Yes/No)** |
|---|---|---|---|
| **Demographics measures** | | | |
| Age (years) | 210 | 11.3 | Yes |
| Sex | 209 | 11.1 | Yes |
| **Behavioural measures** | | | |
| Current smokers | 216 | 11.5 | Yes |
| Alcohol use disorder | 217 | 11.5 | Yes |
| Vaccinated for COVID-19 | 329 | 17.4 | Yes |
| **Medical factors** | | | |
| Obesity | 215 | 11.3 | Yes |
| Hypertension | 213 | 11.3 | Yes |
| Chronic respiratory condition | 5 | 0.27 | Yes |
| Chronic cardiovascular condition | 219 | 11.6 | Yes |
| Diabetes Mellitus | 213 | 11.3 | Yes |
| Chronic kidney disease | 217 | 11.5 | Yes |
| Receiving immunosuppressive medication | 212 | 11.2 | Yes |
| Chronic liver disease | 213 | 11.3 | Yes |
| Malignancy | 215 | 11.4 | Yes |
| Chronic haematological condition | 0 | 0 | No |
| Chronic neurological condition | 0 | 0 | No |
| Number of chronic conditions | 233 | 12.4 | Yes |
| Admitted to the ICU | 0 | 0 | No |
| Received oxygen | 220 | 11.7 | Yes |
| Received antibiotics | 221 | 11.7 | Yes |
| Received corticosteroids | 315 | 16.7 | Yes |
| Received remdesivir | 315 | 16.7 | Yes |
| **Presence of Long COVID Symptoms** | | | |
| Fatigue | 59 | 3 | Yes |
| Dyspnoea | 28 | 1.4 | Yes |

| | | | |
|---|---|---|---|
| Cough | 154 | 8.2 | Yes |
| Chest pain | 148 | 7.8 | Yes |
| Palpitations | 154 | 8.2 | Yes |
| Dizziness | 148 | 7.8 | Yes |
| Joint/muscle pain | 136 | 7.2 | Yes |
| Loss of taste/smell | 99 | 5.2 | Yes |
| Insomnia | 100 | 5.3 | Yes |
| Headache | 155 | 8.2 | Yes |

**Alcohol use disorder**: based on Diagnostic and Statistical Manual of Mental Disorders (DSM–5) criteria.

**Vaccinated for COVID-**19 refers to receipt of any time of COVID-19 before admission irrespective of number of doses during admission.

**Obesity**: body mass index ≥ 30 kg/m2, data collected as a yes or no in the electronic medical records

**Chronic respiratory condition:** Asthma, Alpha 1 trypsin deficiency, Asbestosis, Cryptogenic organizing pneumonia (COP), LAM, LICS, Broncho-pulmonary dysplasia or Primary ciliary dyskinesia, Bronchiectasis, Cystic fibrosis, COPD (chronic bronchitis, emphysema), Lung fibrosis, Sarcoidosis, Obstructive sleep apnoea, Pulmonary hypertension.

**Chronic cardiovascular condition:** Myocardial infarction, Cardiac arrhythmias: AVNRT, Atrial fibrillation, (supra)ventricular tachycardia, Ventricular tachycardia, Brugada syndrome, Sick sinus syndrome, Wolf Parkinson white syndrome, Heart failure, Cardiomyopathy, Valve disease: aortic valve stenosis, aortic valve insufficiency, mitral regurgitation, tricuspid regurgitation, and all other forms of valvular heart disease.

**Diabetes Mellitus**

Diabetes mellitus includes the condition itself plus its complications such as diabetic foot, diabetic polyneuropathy, diabetic retinopathy, diabetic nephropathy.

**Chronic kidney disease:** Acute tubular necrosis or tubulo interstitial nephritis (TIN), Atypical haemolytic uremic syndrome (aHUS), Amyloidosis, Anti-GBM nephritis, Bartter syndrome, Kidney damage due to medication, Chronic bladder infections/kidney infections, Cryoglobulinemia, Cysts, Cystinosis, Dense deposit disease (DDD),Focal segmental glomerulosclerosis (FSGS), Gitelman syndrome,HNF1 beta associated kidney disease, Horseshoe kidneys, IgA nephropathy, Medullary Sponge Kidneys, Membranous nephropathy, Minimal change disease, mononier Nail-patella syndrome (NPS), Nephrogenic diabetes insipidus, Nephronophthisis, Nephrosclerosis, Nephrotic syndrome, Renal angiomyolipoma's, Kidney filter, Primary hyperoxaluria, Reflux nephropathy, Shrivel kidneys scleroderma, SLE nephritis, Alport's syndrome, Systemic Vasculitis received dialysis, a kidney transplant, uremia.

**Receiving Immunosuppressive medication:** Azathioprine, Lenalidomide, Methotrexate, Pirfenidone, Epomalidomide, Thalidomide, Abatacept, Apremilast, Baricitinib, Belatacept, Belimumab, Eculizumab, Vedolizumab, Everolimus, Leflunomide, Mycophenolic acid, Sirolimus, Thymocyte globulin, Tofacitinib, Upadacinitib,

**Chronic haematological condition:** Chronic lymphoblastic leukaemia /Acute leukaemia, Thalassemia, Sickle Cell Disease, Haemolytic Disorders, Clotting disorders (Haemophilia, von Willebrand disease, Thrombophilia).

**Chronic neurological condition:** Amyotrophic lateral sclerosis (ALS), Muscular dystrophies (Duchenne, Becker muscular dystrophy), Multiple sclerosis, Parkinson's disease, Guillain barre with still phenomena, Cerebral vascular accidents, (bloody/non- blooded) /transient ischemic attack, Pompeii disease, Dementia.

**Chronic liver disease:** Cirrhosis, Portal hypertension, Chronic hepatitis.

**Received corticosteroids:** Hydrocortisone, Prednisone, Dexamethasone, Methylprednisolone.

**Received antibiotic medications** applies to all forms of antibiotics.

**Appendix 3.** Association of duration of invasive ventilation with incidence of long COVID (imputed data).

| **Variable** | **Crude model** | **Fully adjusted model** |
|---|---|---|
| | **PR (95% CI)** | **PR (95% CI)** |
| Duration of ventilation (N=385) | 1.007(1.001-1.016) | **1.007(1.000-1.019)** |

**PR** = Prevalence ratios obtained via robust Poisson regression and their 95% confidence intervals. Long COVID/symptoms as outcomes, migration background as the predictor.

**Fully adjusted model =** adjusted for age+ sex+ receiving corticosteroids + smoking + vaccination status against COVID-19 + number of comorbidities + remdesivir therapy.

**Appendix 4.** Differences in patient follow up rates at one year per migration background (imputed data).

| Migration background | Had long COVID at 12 weeks<br><br>N=483 | Participated again at one year<br><br>N=285 | Loss to follow up.<br><br>N (%) | P-value of differences in follow up times between group |
|---|---|---|---|---|
| Dutch origin | 174 | 112 | 62(35.6) | <0.001 |
| African Surinamese origin | 59 | 33 | 26(44.1) | |
| South Asian Surinamese origin | 24 | 17 | 7(29.2) | |
| Moroccan origin | 44 | 22 | 22(50.0) | |
| Turkish origin | 24 | 19 | 5(20.8) | |
| Other origin | 79 | 37 | 42(53.2) | |
| Unknown origin | 79 | 45 | 34(43.0) | |

**Appendix 5.** Clinical predictors of long COVID (unimputed data)

| **Variable** | **Categories** | **Variance Inflation Factor (VIF)** | **Model 1**<br>**PR (95% CI)** |
|---|---|---|---|
| Migration background | | | |
| | Dutch origin | | Reference |
| | African Surinamese origin | | **1.51(1.14-2.01)** |
| | South Asian Surinamese origin | | **1.53(1.02-2.28)** |
| | Moroccan origin | | **1.20(1.01-1.65)** |
| | Turkish origin | | **1.76(1.22-2.55)** |
| | Other origin | | 1.20(0.92-1.57) |
| | Unknown origin | 1.33 | 1.23(0.87-1.63) |
| | | | |
| Age (years) | | 1.28 | 1.00(0.99-1.00) |
| | | | |
| Sex | Male | | Reference |
| | Female | 1.10 | **1.31(1.10-1.58)** |
| | | | |
| Smoking | Never smoked | | Reference |
| | Current smoker | | 1.10(0.90-1.35) |
| | Past smoker | 1.13 | 0.84(0.54-1.34) |
| | | | |
| Alcohol consumption | No | | Reference |
| | Yes | 1.04 | 1.14(0.62-2.10) |
| | | | |
| Vaccinated against COVID-19 | No | | Reference |
| | Yes | 1.08 | 0.83(0.55-1.23) |
| | | | |
| Number of chronic health conditions | None | | Reference |
| | 1 condition | | 0.94(0.72-1.22) |
| | 2 conditions | | 0.93(0.70-1.23) |
| | 3 conditions | | 1.04(0.76-1.41) |
| | 4+ conditions | 1.31 | 1.08(0.79-1.48) |
| | | | |
| Number of days admitted in hospital | | 1.05 | **1.004(1.001-1.01)** |
| | | | |
| Rehospitalization | No | | Reference |
| | Yes | 1.49 | 1.29(0.91-1.83) |
| | | | |
| Admitted to the ICU | No | | Reference |
| | Yes | 1.78 | **1.45(1.14-1.84)** |
| | | | |
| Received oxygen | No | | Reference |
| | Yes | 1.15 | **2.31(1.51-3.53)** |
| | | | |
| Received antibiotics | No | | Reference |
| | Yes | 1.44 | 1.12(0.91-1.38) |
| | | | |
| Received corticosteroids | No | | Reference |
| | Yes | 1.28 | **0.69(0.57-0.84)** |
| | | | |
| Received remdesivir | No | | Reference |
| | Yes | 1.13 | 1.19(0.92-1.53) |
| | | | |

Complete case analysis of clinical predictors of long COVID (i.e., utilization of unimputed data)

**PR** = prevalence ratios with 95% confidence interval obtained via robust Poisson regression.

**Variance inflation factor** (VIF) analysis to identify predictors that are correlated. VIF scores greater than five indicate high correlation between predictors.

**Model 1**: multivariate robust Poisson regression analyses.

**Vaccinated against COVID-**19 refers to receipt of any time of COVID-19 before admission irrespective of number of doses at admission.

**Received corticosteroids:** hydrocortisone, prednisone, dexamethasone, methyl-prednisone.

**Received antibiotic medications** applies to all forms of antibiotics.

**Appendix 6. Unimputed data:** association of migration background with incidence of long COVID

| Long COVID symptoms | (Long COVID per group total) | Crude model | Fully adjusted model |
|---|---|---|---|
| | **N** | **PR (95% CI)** | **PR (95% CI)** |
| **General incidence of long COVID** | | | |
| Dutch origin | 174/769 | 1.00(ref) | 1.00(ref) |
| African Surinamese origin | 59/177 | 1.49(1.16-1.89) | **1.49(1.12-1.96)** |
| South Asian Surinamese origin | 24/72 | 1.47(1.04-2.09) | **1.50(1.01-2.24)** |
| Moroccan origin | 44/144 | 1.35(1.02-1.78) | **1.21(1.01-1.77)** |
| Turkish origin | 24/67 | 1.66(1.18-2.33) | **1.72(1.20-2.46)** |
| Other origin | 79/310 | 1.13(0.90-1.43) | 1.20(0.92-1.56) |
| Unknown origin | 79/340 | 1.05(0.84-1.33) | 1.21(0.92-1.60) |

The migration background groups with one- or no-person reporting symptoms of heart palpitations, dizziness, joint and muscular discomfort, and loss of taste and smell were excluded from this table.

**PR** = Prevalence ratios obtained via robust Poisson regression and their 95% confidence intervals. Long COVID/symptoms as outcomes, migration background as the predictor.

**Fully adjusted model**: adjusted for age + sex + statistically significant determinants of long COVID (i.e., number of days admitted to hospital + admission to ICU + receiving oxygen therapy + receiving steroid therapy) + other non-statistically significant determinants of long COVID (i.e., smoking + vaccination status against COVID-19 + number of comorbidities + receiving remdesivir therapy).

Interactions between oxygen therapy and steroid therapy, as well as between admission to ICU and steroid therapy were not statistically significant hence not included as additional effects in the models.

**Appendix 7. Various multiple imputations datasets:** association of migration background with incidence of long COVID and individual long COVID symptoms

| General incidence of long COVID | (Long COVID per group total) | Crude Model | Fully adjusted model |
|---|---|---|---|
| | N | PR (95% CI) | PR (95% CI) |
| **Imputed dataset 2** | | | |
| Dutch origin | 174/776 | 1.00(ref) | 1.00(ref) |
| African Surinamese origin | 59/177 | 1.48(1.16-1.89) | **1.45(1.13-1.87)** |
| South Asian Surinamese origin | 24/72 | 1.47(1.04-2.09) | **1.56(1.09-2.23)** |
| Moroccan origin | 44/144 | 1.35(1.02-1.78) | **1.34(1.01-1.78)** |
| Turkish origin | 24/67 | 1.66(1.18-2.33) | **1.51(1.09-2.10)** |
| Other origin | 81/310 | 1.13(0.90-1.43) | 1.12(0.89-1.41) |
| Unknown origin | 79/340 | 1.05(0.84-1.33) | 1.11(0.88-1.41) |
| **Imputed dataset 3** | | | |
| Dutch origin | 127/776 | 1.00(ref) | 1.00(ref) |
| African Surinamese origin | 39/177 | 1.48(1.16-1.89) | **1.43(1.11-1.84)** |
| South Asian Surinamese origin | 15/72 | 1.47(1.04-2.09) | **1.54(1.07-2.21)** |
| Moroccan origin | 29/144 | 1.35(1.02-1.78) | **1.33(1.01-1.77)** |
| Turkish origin | 12/67 | 1.66(1.18-2.33) | **1.54(1.09-2.12)** |
| Other origin | 52/310 | 1.13(0.90-1.43) | 1.12(0.89-1.42) |
| Unknown origin | 66/340 | 1.05(0.84-1.33) | 1.09(0.86-1.38) |
| **Imputed dataset 4** | | | |
| Dutch origin | 112/776 | 1.00(ref) | 1.00(ref) |
| African Surinamese origin | 32/177 | 1.48(1.16-1.89) | **1.47(1.14-1.88)** |
| South Asian Surinamese origin | 14/72 | 1.47(1.04-2.09) | **1.56(1.08-2.23)** |
| Moroccan origin | 22/144 | 1.35(1.02-1.78) | **1.33(1.01-1.77)** |
| Turkish origin | 16/67 | 1.66(1.18-2.33) | **1.56(1.08-2.23)** |
| Other origin | 45/310 | 1.13(0.90-1.43) | 1.12(0.88-1.42) |
| Unknown origin | 67/340 | 1.05(0.84-1.33) | 1.12(0.88-1.42) |
| **Imputed dataset 5** | | | |
| Dutch origin | 26/776 | 1.00(ref) | 1.00(ref) |
| African Surinamese origin | 8/177 | 1.48(1.16-1.89) | **1.43(1.11-1.84)** |
| South Asian Surinamese origin | 5/72 | 1.47(1.04-2.09) | **1.56(1.08-2.23)** |
| Moroccan origin | 7/144 | 1.35(1.02-1.78) | 1.32(1.00-1.75) |
| Turkish origin | 6/67 | 1.66(1.18-2.33) | **1.54(1.07-2.21)** |
| Other origin | 11/310 | 1.13(0.90-1.43) | 1.11(0.88-1.40) |
| Unknown origin | 10/340 | 1.05(0.84-1.33) | 1.11(0.88-1.40) |

The migration background groups with one- or no-person reporting symptoms of heart palpitations, dizziness, joint and muscular discomfort, and loss of taste and smell were excluded from this table.

**PR** = Prevalence ratios obtained via robust Poisson regression and their 95% confidence intervals. Long COVID/symptoms as outcomes, migration background as the predictor.

**Fully adjusted model**: adjusted for age + sex + statistically significant determinants of long COVID (i.e., number of days admitted to hospital + admission to ICU + receiving oxygen therapy + receiving steroid therapy) + other non-statistically significant determinants of long COVID (i.e., smoking + vaccination status against COVID-19 + number of comorbidities + receiving remdesivir therapy).

Interactions between oxygen therapy and steroid therapy, as well as between admission to ICU and steroid therapy were not statistically significant hence not included as additional effects in the models.
